# Relationship between gut microbiota and lung function decline in patients with chronic obstructive pulmonary disease: a 1-year follow-up study

**DOI:** 10.1186/s12931-022-01928-8

**Published:** 2022-01-15

**Authors:** Yu-Chi Chiu, Shih-Wei Lee, Chi-Wei Liu, Tzuo-Yun Lan, Lawrence Shih-Hsin Wu

**Affiliations:** 1 Department of Internal Medicine, Taoyuan General Hospital, Ministry of Health and Welfare, Taoyuan, Taiwan; 2 Institute of Hospital and Health Care Administration, National Yang Ming Chiao Tung University, Taipei, Taiwan; 3 Department of Nursing, Yuanpei University of Medical Technology, Hsinchu, Taiwan; 4 Graduate Institute of Biomedical Sciences, China Medical University, No. 91 Hsueh-Shih Road, Taichung, 404 Taiwan; 5 Center of Allergy, Immunology, and Microbiome (A.I.M.), China Medical University Children’s Hospital, Taichung, Taiwan

**Keywords:** Chronic obstructive pulmonary disease, Stool sample, Next generation sequencing, Gut microbiota, Lung function decline

## Abstract

**Objective:**

Chronic obstructive pulmonary disease (COPD) is a chronic inflammatory lung disease characterized by a persistent limitation in airflow. Gut microbiota is closely correlated with lung inflammation. However, gut microbiota has not been studied in patients with declining lung function, due to chronic lung disease progression.

**Subjects and methods:**

Stool samples were obtained from 55 patients with COPD that were in stable condition at enrolment (stage 1) and at a 1-year follow-up (stage 2). After extracting stool DNA, we performed next generation sequencing to analyse the distribution of gut microbiota.

**Results:**

Patients were divided to control and declining lung function groups, based on whether the rate of forced expiratory volume in 1 s (FEV_1_) had declined over time. An alpha diversity analysis of initial and follow-up stool samples showed a significant difference in the community richness of microbiota in the declining function group, but not in the control group. At the phylum level, *Bacteroidetes* was more abundant in the control group and *Firmicutes* was more abundant in the declining function group. The *Alloprevotella* genus was more abundant in the control group than in the declining function group. At 1-year follow-up, the mean proportions of *Acinetobacter* and *Stenotrophomonas* significantly increased in the control and declining function groups, respectively.

**Conclusion:**

Some community shifts in gut microbiota were associated with lung function decline in COPD patients under regular treatment. Future studies should investigate the mechanism underlying alterations in lung function, due to changes in gut bacterial communities, in COPD.

**Supplementary Information:**

The online version contains supplementary material available at 10.1186/s12931-022-01928-8.

## Background

Chronic obstructive pulmonary disease (COPD) is the fourth leading cause of death worldwide. It affects approximately 10% of individuals older than 45 years [1, 2].This chronic, slowly progressive disease leads to irreversible airway obstruction [3]. COPD is associated with chronic inflammation of the airways and lung parenchyma and also with systemic inflammation [4]. In COPD, chronic inflammation is characterized by elevations in alveolar macrophages, neutrophils, T lymphocytes, and innate lymphoid cells, which secrete a variety of proinflammatory mediators, including cytokines, chemokines, growth factors, and lipid mediators [5].

Various inflammatory pathways in lung diseases are regulated by the human microbiota [6]. Previous studies have indicated that gut microbiota stimulated the production of reactive oxygen species in alveolar macrophages [7], which can drive COPD progression [8].

Patients with COPD frequently have other comorbidities, such as cardiovascular disease (CVD) [9], lung cancer [10], diabetes [11], metabolic syndrome [12], malnutrition [13], osteoporosis [14], anxiety, and depression [15]. These comorbidities may influence disease severity and survival. Smoking and aging are well-known risk factors in COPD [16]. Bacterial colonization in the lung has been associated with exacerbations and loss of lung function in patients with COPD [17, 18]. Moreover, the lung microbiota plays an important role in COPD development [19, 20].

Interestingly, studies on ulcerative colitis and Crohn’s disease have suggested that the ‘gut-lung axis’ may be important in COPD pathogenesis [21, 22]. However, the characteristics of gut microbiota in patients with COPD are not fully understood. Previous studies have shown community shifts in gut microbiota, in response to cigarette smoking [23, 24], which suggested that there is some immunological coordination between the lungs and the gut. Despite the importance of gut microbiota in COPD, it remains unknown how gut microbiota might be associated with lung function decline in COPD.

The distributions of respiratory microbiota are significantly different between healthy individuals and individuals with COPD, and between patients with different levels of COPD severity [19]. Recent interest in the influence of probiotics on asthma and COPD [25] has spurred investigations into whether the gut microbiota is related to COPD exacerbation or severity. Indeed, patients with COPD have different faecal microbiota and metabolomes, compared to healthy subjects [26]. Moreover, we previously showed that the gut microbiota was associated with COPD severity [27]. In the present study, we aimed to evaluate changes in gut microbiota during 1 year of lung function decline in patients with COPD who have regular therapy.

## Patients and methods

### Patients

We enrolled 55 patients over 40 years old with COPD. COPD diagnoses and classifications were established according to recommendations from the Global Initiative for Chronic Obstructive Lung Disease (GOLD) [28]. The spirometry tests were conducted by using the Platinum Elite Series™ body plethysmograph (MGC Diagnostics Corporation, Saint Paul, MN, USA) according to the recommendations of the European Respiratory Society and the American Thoracic Society [29]. After satisfactory baseline measurements, subjects repeated the spirometry tests 15 min after bronchodilator inhalation (BEROTEC N 200 mcg). As aforementioned, spirometry tests after bronchodilator is required to establish a diagnosis of COPD and determine the classification of COPD severity, based on the presence of FEV_1_/FVC < 0.7 and FEV_1_ (stage 1: mild ≥ 80% predicted; stage 2: moderate 50–79% predicted; stage 3: severe 30–49% predicted; stage 4: very severe < 30% predicted). About the smoking situation, 16 (9 in control group and 7 in decline group) of the subjects were current smokers, 33 (15 in control group and 18 in decline group) former smokers and 6 (3 in control group and 3 in decline group) never smokers (Table 1). We excluded patients diagnosed with cancer, other immune-related diseases, or viral infections (e.g., hepatitis B, hepatitis C, human immunodeficiency virus, etc.). A previous study estimated that, over a 3-year period, the forced expiratory volume in 1 s (FEV_1_) declined by more than 40 ml per year in slightly more than one in three individuals with COPD (38%) [30]. Based on that finding, we defined lung function decline (the decline group) as an FEV_1_ decline greater than 40 ml in 1 year. For the control group, we included patients with COPD that showed FEV_1_ declines less than 40 ml per year and a positive rate of change of ≥ 0 ml per year (i.e., no improvement).

**Table 1:** The clinical characteristics of control and decline groups

Variables	Control group	Decline group	*p* value
Male (n)	27	28	
Age (years)			
Mean ± SD (range)	70 ± 10 (50–89)	74 ± 9 (51–90)	0.105^a^
BH (m)			
Mean ± SD (range)	1.66 ± 0.06 (1.50–1.77)	1.63 ± 0.07 (1.50–1.77)	0.238^a^
BW (kg)			
Mean ± SD (range)	62 ± 11 (43–81)	60 ± 9 (40–75)	0.442^a^
BMI			
Mean ± SD (range)	22.62 ± 3.78 (14.88–29.72)	22.50 ± 3.45 (15.96–29.14)	0.901^a^
Smoking (n)			
Current smoker	9	7	0.777^c^
Former smoker	15	18	
Never smoker	3	3	
WBC (per µl)			
Median (range)	7060 (2580–14,410)	6980 (4090–19,590)	0.685^b^
Eosinophil (%)			
Median (range)	1.9 (0.0–36.1)	2.1 (0.2–10.9)	0.246^b^
Eosinophil (per µl)			
Median (range)	133 (0–4166)	166 (19–701)	0.229^b^
Stages (n)			
Stage 1	8	14	0.111^c^
Stage 2	12	12	
Stage 3	7	2	
CAT			
Mean ± SD	9.93 ± 6.68	8.68 ± 6.02	0.470^a^
Score < 10 (n)	14	19	0.226^c^
Score ≧ 10 (n)	13	9	
mMRC			
Mean ± SD	0.96 ± 0.98	0.89 ± 1.17	0.810^a^
Score < 2 (n)	17	21	0.334^c^
Score ≧ 2 (n)	10	7	
AE (n)			
Yes	7	3	0.144^c^
No	20	25	
Medication (n)			
LABA	1	2	0.175^c^
LAMA	5	4	
LAMA + LABA	7	10	
ICS + LABA	7	1	
ICS + LAMA + LABA	7	11	

*SD* standard deviation, *COPD* chronic obstructive pulmonary disease, *n* number of subjects, *BH* body height, *WB* body weight, *BMI* body mass index, *WBC* white blood cell, *AE* acute exacerbation, *CAT* COPD Assessment Test, *mMRC* Modified Medical Research Council, *FVC* forced vital capacity, *FEV*_*1*_ forced expiratory volume in one second, *LAMA* long-acting muscarinic antagonist, *LABA* long-acting beta agonist, *ICS* inhaled corticosteroid, ^a^The statistical analysis was tested by *t* test; ^b^The statistical analysis was tested by Mann–Whitney test; ^c^The statistical analysis was tested by *χ*^2^-test

We obtained stool samples from patients with COPD in stable condition (i.e., without exacerbations or the use of antibiotics for any other reason for at least 3 months prior). Samples were acquired at the time of enrolment (first sampling, stage 1) and after a 1-year follow-up (second sampling, stage 2).

### Bacterial DNA purification from stool samples

According to the methods of Chi et al*.* [27], bacterial DNA was purified from stool samples collected from the 55 subjects using the AllPure Micro Genomic DNA Extraction Kit (Cat. No. ABTGNA022-50, AllBio Science, Inc., Taichung, Taiwan). Briefly, the 0.5 ~ 1 g stool sample placed in a 15-ml centrifuge tube with 3 ml sterile distilled water and then was mixed thoroughly by vortexing or pipetting. The tubes were centrifuged at 40×*g* (500 rpm) for 30 s, 362×*g* (1000 rpm) for 1 min, and 1449×*g* (3000 rpm) for 1 min, respectively. The supernatants were stored at − 20 °C until DNA extraction.

1 ml of the supernatant was transferred to a 1.5 microcentrifuge tube and then centrifuged at 13,800×*g* (12,000 rpm) for 5 min and discard the supernatant. The pellet was mixed with 200 μl of LB14 buffer and 20 μl of Proteinase K and then incubated at 55 °C for 30 min to lyse the pellet. After centrifugation at 13,800×*g* (12,000 rpm) for 5 min at 4 °C, the supernatant was transferred to a new 1.5 microcentrifuge tube and mixed with 20 μl of RNaseA and incubated at room temperature for 2 min. After mixing with 500 μl of binding buffer, the supernatant was incubated at 55 °C for 10 min and then applied to a Genomic Spin Column for DNA purification and washed twice with clean and wash buffers. DNA was eluted from the spin column with 40 μl of elution buffer (preheated to 65 °C) for a 5-min incubation at room temperature before centrifugation at 13,800×*g* (12,000 rpm) for 2 min. The DNA extraction was performed according to the instructions of AllPure Micro Genomic DNA Extraction Kit. The quality of the extracted DNA was visualized by performing 1% agarose gel electrophoresis and then stored at − 80 °C until NGS analysis.

### MetaVx™ library preparation and illumina MiSeq sequencing

Next generation sequencing (NGS) library preparations and Illumina MiSeq sequencing were conducted at AllBio Science, Inc. (Taichung, Taiwan). After DNA quantification with a Qubit 2.0 Fluorometer (Invitrogen, Carlsbad, CA, USA), 30–50 ng DNA was used to generate amplicons with a MetaVx™ Library Preparation kit (GENEWIZ, Inc., South Plainfield, NJ, USA).

The V3-V4 hypervariable regions of prokaryotic 16S rDNA were selected for generating amplicon libraries and subsequent taxonomy analyses. The V3-V4 hypervariable regions were amplified with forward primers that contained the sequence “CCTACGGRRBGCASCAGKVRVGAAT” and reverse primers that contained the sequence “GGACTACNVGGGTWTCTAATCC”, designed by GENEWIZ. Products from the 1st PCR round were used as templates in a 2nd PCR round for amplicon enrichment. Indexed libraries for downstream NGS sequencing on Illumina Miseq were prepared by adding indexed adapters to the ends of the 16S rDNA amplicons.

DNA libraries were validated and quantified with the Agilent 2100 Bioanalyzer (Agilent Technologies, Palo Alto, CA, USA) and the Qubit 2.0 Fluorometer, respectively. According to manufacturer instructions (Illumina, San Diego, CA, USA), the DNA libraries were multiplexed, then loaded on an Illumina MiSeq instrument. DNA libraries were sequenced in the 2 × 300 paired-end configuration; image analysis and base calling were performed with MiSeq Control Software provided with the MiSeq instrument.

### Data analysis

16S rRNA data was analysed with the quantitative insights into microbial ecology (QIIME) data analysis package. Forward and reverse reads were joined and assigned to samples, based on barcodes, then the barcode and primer sequences were removed. To improve the quality of the joined sequences, we discarded sequences that did not fulfil the following criteria: sequence length < 200 bp, no ambiguous bases, and a mean quality score ≥ 20. Then, the sequences were compared to the reference database (RDP Gold database). Chimeric sequences were detected and removed with the UCHIME algorithm (https://drive5.com/uchime/uchime_download.html). The clustering program, VSEARCH (1.9.6) [31], was applied to the Silva 119 database, pre-clustered at 97% sequence identity, to group the effective sequences into operational taxonomic units (OTUs). The Ribosomal Database Program (RDP) classifier was applied with a confidence threshold of 0.8 to assign taxonomic categories to all OTUs. The RDP classifier was applied to the Silva 132 database to assign species-level classifications.

The alpha and beta diversity statistics of amplicon sequencing data were rarefied prior to calculations. In alpha diversity analyses, the Chao1, Shannon, and Simpson indices were calculated in QIIME (version 1.9.1) [32] from rarefied samples. In beta diversity analyses, R version 3.1.1 (https://cran.r-project.org/bin/windows/base/old/ 3.1.1/) was used to perform principal component analyses and plots, based on the Brary-Curtis distance matrix. The ecological and heatmap analyses were performed with the pheatmap package (https://cran.r-project.org/src/contrib/Archive/pheatmap/). Differences in taxonomic composition at the genus level were evaluated between different COPD groups with Metastats. Differences in the abundances of microbial communities were evaluated between the groups with strict statistical methods. Significant differences were determined with the multiple hypothesis test and the false discovery rate (FDR) in the rare frequency data. FDR-adjusted p-values were calculated with the Benjamini–Hochberg procedure. Differences in strain abundance between groups were analysed with Statistical Analysis of Metagenomic Profiles (STAMP) software, and Welch’s t-test was the default setting for two-group comparisons.

## Results

### Demographic and clinical features

We enrolled 55 patients with COPD that completed a 1-year follow-up (Table 1). Patients were divided into control (n = 27) and decline (n = 28) groups, based on whether their pre-bronchodilator FEV_1_ declined less or more than 40 ml/year, respectively. All demographic and clinical features were similar between groups.

**Fig. 1:**
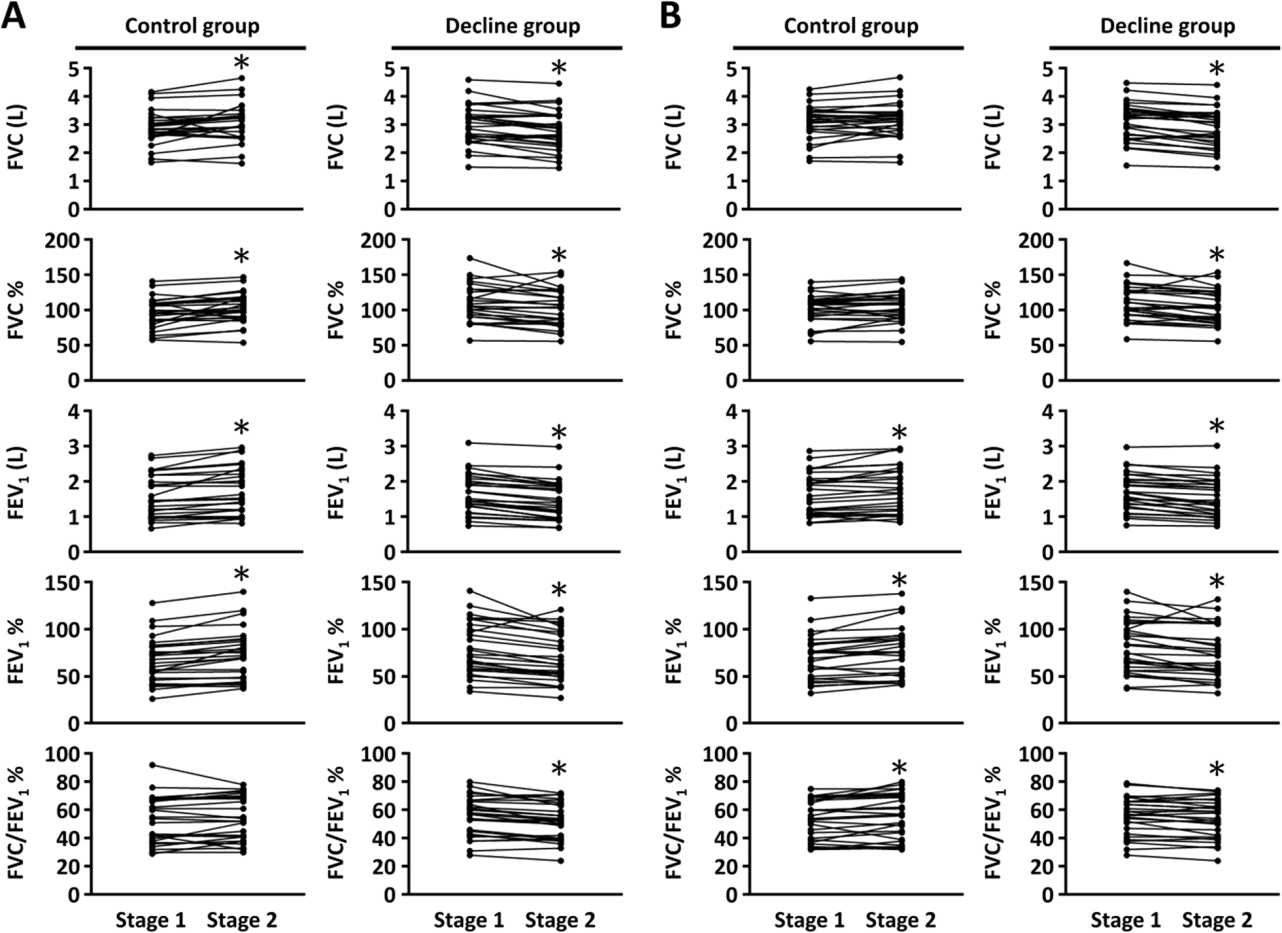
The alteration of lung function parameters in each COPD patient. **A** pre-bronchodilator; **B** post-bronchodilator. *p < 0.05, paired *t* test; stage 1: lung function measurement, first time; stage 2: lung function measurement, 1-year after stage 1

The lung function parameters were not significantly different between control and decline groups in stage 1 or stage 2 (Fig. 1), regardless of whether the measurements were performed pre- or post-bronchodilator (Additional file 1: Table S1). However, most lung function parameters changed significantly between stage 1 and stage 2, in both the control and decline groups (Fig. 1). The average parameter values increased in the control group and decreased in the decline group between stages 1 and 2 (Additional file 1: Table S1). In particular, the pre-bronchodilator FEV_1_ decreased in the decline group and increased in the control group (Fig. 1).

### Alterations in alpha and beta diversity

**Fig. 2:**
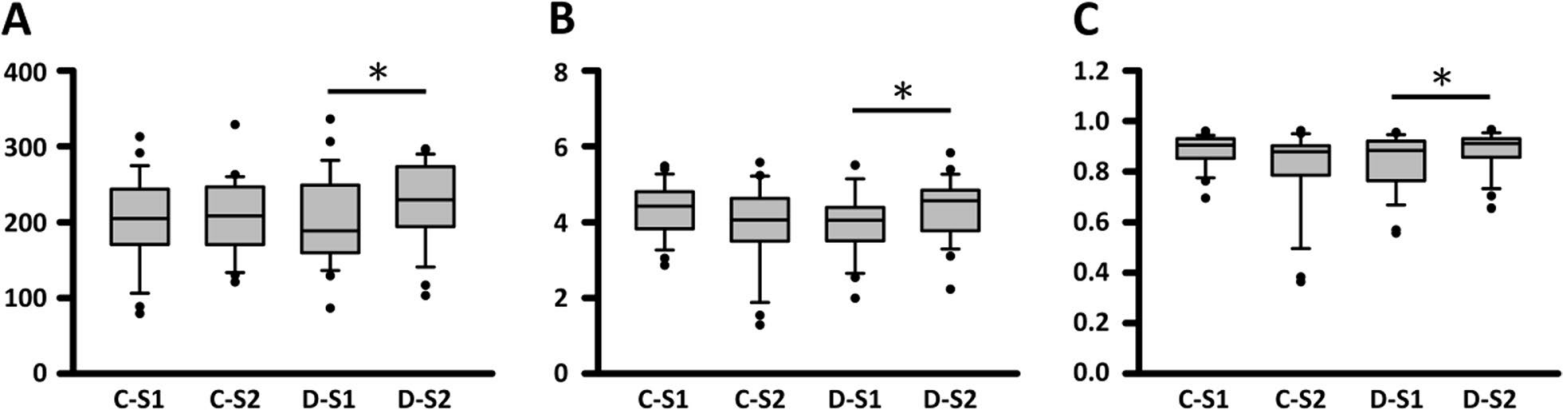
The indices for community richness (**A** Chao 1 index) and diversity (**B** and **C** Shannon and Simpson indices) of four COPD groups. *p < 0.05, Wilcoxon test. C: control; D: decline; S1: stage 1; S2: stage 2

We evaluated the Chao1, Shannon, and Simpson indices to estimate the alpha diversity of gut microbiota in stage 1 and stage 2 samples (Fig. 2A–C). The OTU richness and diversity indices were not significantly different between the two stages in the control group. However, all three indices increased significantly from stage 1 to stage 2 in the decline group.

In the beta diversity analysis, we found no clusters in any of these four COPD groups (Additional file 1: Fig. S1).

### Taxonomic distributions

**Fig. 3:**
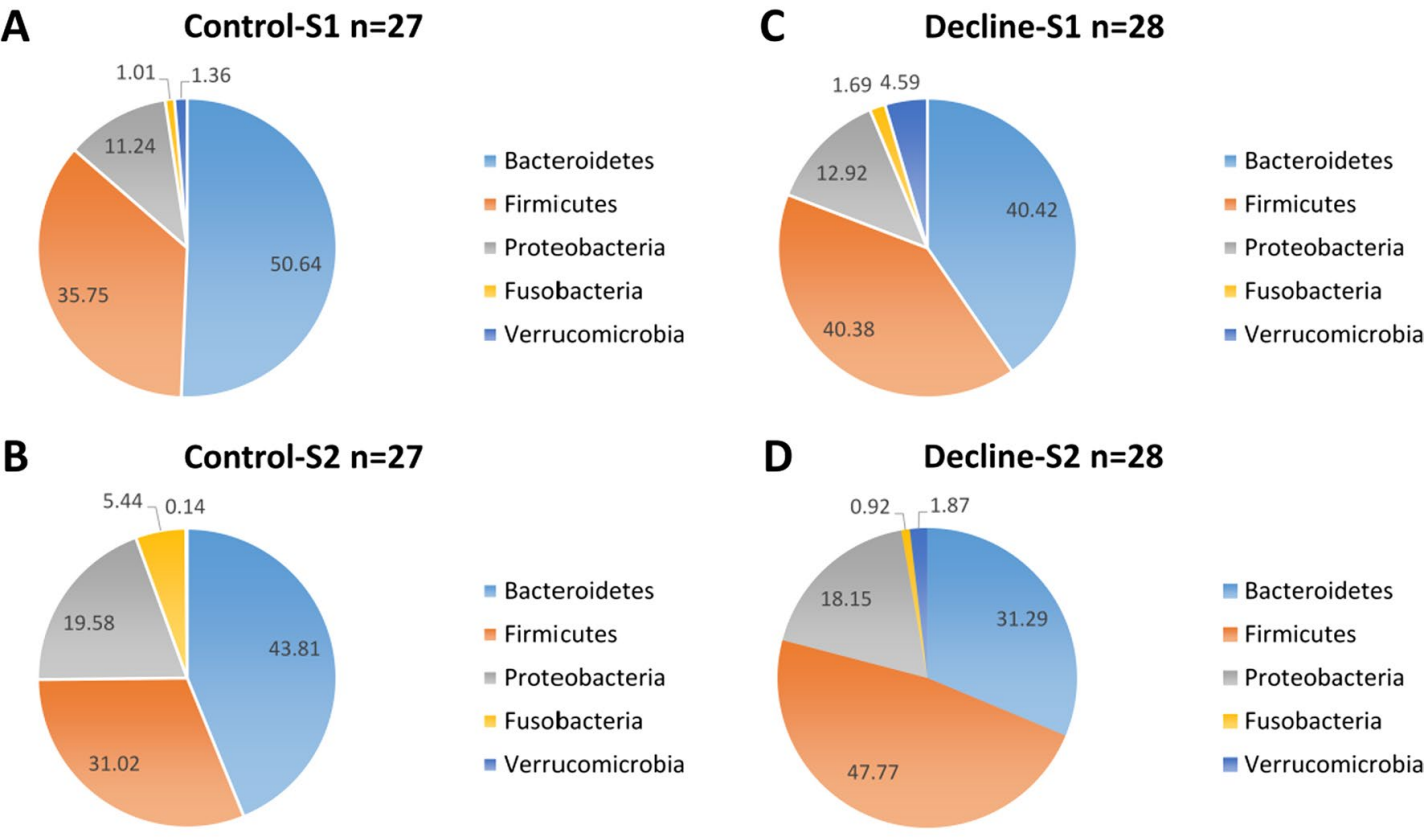
The distribution of five most abundant phyla (*Firmicutes*, *Bacteroidetes*, *Proteobacteria*, *Fusobacteria*, and *Verrucomicrobia*) in different COPD groups. **A**: Control-S1; **B**: Control-S2; **C**: Decline-S2; **D**: Decline-S2

At the phylum level, *Bacteroidetes* was more abundant in the control group and *Firmicutes* was more abundant in the decline group (Fig. 3). At the 1-year follow-up, the proportion of *Firmicutes* decreased in the control group and increased in the decline group (Fig. 3).

At the genus level, the phylogenetic tree of OTUs (Additional file 1: Fig. S2) showed that the 30 most abundant OTUs across all samples belonged to five major phyla (Fig. 3). We plotted heatmaps of the 30 most abundant taxa, identified at the genus or species level, in each study group (Additional file 1: Fig. S3). The group cluster analysis, based on Euclidean distance, showed that the similarity between stages 1 and 2 was higher in the control group than in the decline group. Moreover, the genera in the stage 1 decline group clustered with the genera in the control group (Additional file 1: Fig. S3A). In particular, in the decline group, *Tyzzerella 4* was more abundant and *Prevotella_9* was less abundant in stage 2 than in stage 1 (Additional file 1: Fig. S3A). In the analysis at the species level (Additional file 1: Fig. S3B), many OTUs were not identified (unclassified).

### Differential abundance

The differential abundance analysis was performed at the genus level. We evaluated the relative abundances of the five genera that showed the largest between-group differences (Table 2). In both stages 1 and 2, *Alloprevotella* was more abundant in the control group than in the decline group. However, some of the statistical significance might have been due to the contributions of outliers (Additional file 1: Fig. S4C, D).

**Table 2:** The differences in abundance distributions of the five genera with the largest between-group differences

Comparison	Phylum	Genus	Increase (+)/Decrease (−)^#^
Control-S1 *vs*	*Fusobacteria*	*Cetobacterium* ***	+
Control-S2	*Firmicutes*	*Lachnospiraceae_NK4A136_group* *	−
	*Proteobacteria*	*Pluralibacter* ***	−
	*Firmicutes*	*Ruminococcaceae_NK4A214_group* *	+
	*Firmicutes*	*Tyzzerella*_4 *	−
Decline-S1 *vs*	*Proteobacteria*	*Citrobacter* *	+
Decline-S2	*Firmicutes*	*[Eubacterium]_eligens_group* **	+
	*Firmicutes*	[Eubacterium]_ventriosum_group**	−
	*Bacteroidetes*	*Prevotella*_2 **	−
	*Firmicutes*	*Weissella* **	+
Decline-S1 *vs*	*Bacteroidetes*	Alloprevotella***	+
Control-S1	*Firmicutes*	[Eubacterium]_ventriosum_group*	−
	*Proteobacteria*	Pluralibacter***	+
	*Bacteroidetes*	Prevotellaceae_NK3B31_group***	+
	*Firmicutes*	*Ruminiclostridium*_6*	+
Decline-S2 *vs*	*Firmicutes*	Acidaminococcus**	−
Control-S2	*Bacteroidetes*	Alloprevotella***	+
	*Actinobacteria*	Bifidobacterium***	−
	*Fusobacteria*	Cetobacterium***	+
	*Firmicutes*	Weissella***	−

^#^Reference group: the front group; *p < 0.05; **p < 0.01; ***p < 0.001, Fisher’s exact test

**Fig. 4:**
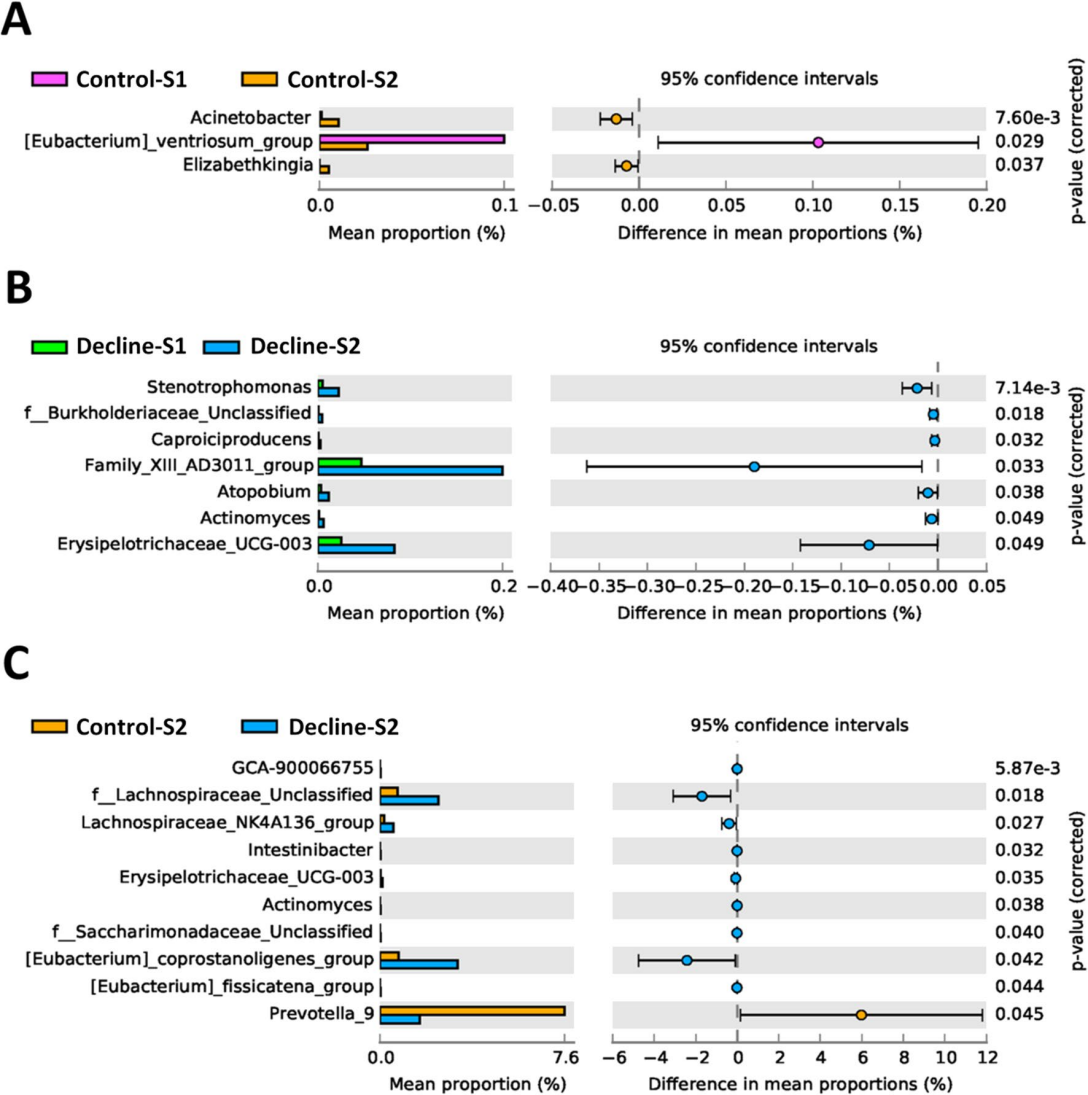
STAMP analysis at the genus levels showed significant differences in mean proportion between different COPD groups. The statistical analysis was tested by Welch’s t-test. **A**: Control-S1 vs. Control-S2; **B**: Decline-S1 vs. Decline-S2; **C**: Control-S2 vs. Decline-S2

We compared strain abundances between groups at the genus level (Fig. 4). Significant increases in the mean proportions of *Acinetobacter* and *Stenotrophomonas* were observed after 1-year in the control and decline groups, respectively (Fig. 4A, B). At stage 1, the mean proportions were not significantly different between control and decline groups (data not shown). However, at stage 2, the mean proportions of ten OTUs (genera) were significantly different between control and decline groups (Fig. 4C).

## Discussion

Although COPD is considered a progressive disease, only some patients display an estimated FEV_1_ decline greater than 40 ml per year [30]. In this study, we found that bacterial richness and diversity in stool samples changed significantly when the FEV_1_ declined more than 40 ml/year (decline group). Moreover, the most abundant phyla were different between the control (*Bacteroidetes*) and decline (*Firmicutes*) groups. Furthermore, both before and after the 1-year follow-up, the *Alloprevotella* genus was more abundant in the control group than in the decline group. At the 1-year follow-up, the mean proportions of the *Acinetobacter* and *Stenotrophomonas* genera significantly increased in the control and decline groups, respectively.

Previous studies have shown that current smoking was strongly associated with the rate of FEV_1_ decline [30]. Compared to non-smokers, the gut microbiota in current smokers exhibited more abundant *Bacteroidetes* and less abundant *Firmicutes* and *Proteobacteria* [33]. At the genus level, the gut microbiota of smokers harboured more *Bacteroides* and *Prevotella* compared to that of non-smokers [34]. Compared to continuing smokers and non-smokers, gut microbiota of ex-smokers that had returned to smoking exhibited more overall microbial diversity and harboured larger proportions of *Firmicutes* and *Actinobacteria*, and smaller proportions of *Bacteroidetes* and *Proteobacteria* plyla [24]. We found that, among patients with COPD, *Bacteroidetes* was more abundant when lung function showed little or no decline (control group) and that *Firmicutes* was more abundant when lung function declined.

In a previous study, 14% of patients with COPD fulfilled the Rome II criteria for irritable bowel syndrome (IBS) [35]. Moreover, Mokhlesi et al*.* found that, among patients with COPD, the prevalence of gastroesophageal reflux symptoms tended to be higher in those with an FEV_1_ > 50% of predicted, compared to those with an FEV_1_ < 50% of predicted [36]. Furthermore, the coexistence of COPD and small intestinal bacterial overgrowth was associated with changes in microbiota that resembled the instability and reduced diversity frequently observed in post-infectious IBS [37, 38]. In the present study, the decline group showed significant increases in the alpha diversity indices in stage 2 compared to stage 1. This finding suggested that the gut microbiota was more unstable in the decline group than in the control group. Future studies should investigate potential associations between gastrointestinal disorders, such as IBS and functional dyspepsia, and lung function decline in COPD.

We found a significant increase in the mean proportion of *Acinetobacter* after 1 year in the control group. A previous study that investigated food introduced in the first year of life and subsequent asthma development showed that unbalanced meat consumption fostered growth of iron scavenging bacteria, such as *Acinetobacter*, which was related to asthma [39]. However, the biological mechanism remains unknown. It is possible that the increase in *Acinetobacter* that we observed in the control group after 1 year might have been due to food consumption.

In the decline group, the mean proportion of *Stenotrophomonas* significantly increased after 1 year. *Stenotrophomonas maltophilia* colonies are commonly found in the respiratory tracts of patients with chronic lung disease [40]. Indeed, some community-acquired pneumonia cases are caused by Gram-negative bacteria, particularly *Pseudomonas aeruginosa*, *Klebsiella pneumoniae*, *Acinetobacter baumannii*, and *Stenotrophomonas maltophilia* [41, 42]. However, we did not detect *Acinetobacter baumannii* or *Stenotrophomonas maltophilia* in stool samples from patients with COPD. Furthermore, it remains unknown whether increases in the proportions of *Acinetobacter* and *Stenotrophomonas* in gut are related to COPD progression.

We also found a reduction in the relative abundance of *Prevotella_2* after 1 year in the decline group (Table 2). Moreover, at stage 2, the control group had a higher mean proportion of *Prevotella_9* than the decline group (Fig. 4). A previous study showed an association between lung microbiota and COPD severity (defined by computed tomography). They found that patients with mild COPD had higher abundances of *Prevotella* in lung samples than patients with severe COPD [43]. In another study, LPS/elastase-treated mice had reduced representation of *Prevotella* in the lung [44]. In gut, *Prevotella* strains are associated with plant-rich diets, but they are also linked with chronic inflammatory conditions [45]. Based on those findings, we suggest that the abundance of *Prevotella* might be associated with a putative COPD subtype that is related to the rate of lung function decline.

The microbiota could be used as potential markers for the progression of HBV-related acute-on-chronic liver failure [46] and the management of cancer [47]. Previous studies indicated that gut microbiota regulated lung inflammation [48] and was associated with respiratory diseases [49]. In addition, in mice model, gut microbiota from patients with COPD induced lung inflammation and adaptive immune responses [50]. The increased ratio of Firmicutes/Bacteroidetes species as well as segmented filamentous bacteria colonization is associated with lung IL-17 and IL-22 responses and enhances airway hyperresponsiveness [48]. The increased Bacteroidetes and Actinobacteria species decrease airway inflammation by the production of short chain fatty acids and regulation of Treg cells [48]. Whether any immune molecules and cells are involved in the lung function decline caused by the intestinal microbiota needs further investigation.

Our study had some limitations. First, we had relatively small COPD groups, which could have limited our statistical power. Future studies are necessary, with larger groups, to confirm our findings. Second, we mostly analysed microbiota genera, rather than species. Therefore, we could not infer the roles of certain species, particularly pathogens, in COPD progression. Finally, in this study, we chosen the definition of decline group (FEV_1_ declined more than 40 ml/year) according to previous study [30] and because the annualized absolute loss of FEV_1_ of ≧ 40 ml/year was a significant predictor for incident COPD risk among ever smokers without prevalent obstructive and nonobstructive lung disease at baseline examination [51]. However, a recent report indicated that year-to-year changes in FEV_1_ should exceed 15% before considering it as a clinically meaningful change in patients with COPD [52]. In this study, 11 of decline subjects were fit the criteria year-to-year FEV_1_ variability exceeded 15%. In future study, it would be worthwhile to evaluate the difference in gut microbiota between subjects whose losses year-to-year FEV_1_ exceed 40 ml and 15%.

In conclusion, we found that community shifts in gut microbiota were associated with lung function decline. Our results suggested that the gut microbiota might influence COPD progression.

## Supplementary Information

**Additional file 1.** **Supplemental Table 1.** Comparison of lung function parameters for control and decline groups. **Supplemental Fig. 1.** Plot of principal component analysis (PCA) for different COPD groups. S1: stage 1; S2: stage 2. **Supplemental Fig. 2.** The phylogenic tree of the 30 most abundant OTUs across the samples at the genus level. **Supplemental Fig. 3.** The heatmap analysis of the top 30 OTUs. A: At genus level. B: At species level.

## Data Availability

The data of 16S rRNA V3 + V4 hypervariable regions by sequencing for stool from COPD patients had submitted to European Nucleotide Archive, Accession No: PRJEB43280.

## Abbreviations

SD: Standard deviation
COPD: Chronic obstructive pulmonary disease
BH: Body height
WB: Body weight
BMI: Body mass index
WBC: White blood cell
AE: Acute exacerbation
CAT: COPD assessment test
mMRC: Modified Medical Research Council
FVC: Forced vital capacity
FEV_1_: Forced expiratory volume in one second
LAMA: Long-acting muscarinic antagonist
LABA: Long-acting beta agonist
ICS: Inhaled corticosteroid
OTU: Operational taxonomic unit
GOLD: Global initiative for chronic obstructive lung disease
NGS: Next generation sequencing
QIIME: Quantitative insights into microbial ecology
RDP: Ribosomal database program
FDR: False discovery rate
STAMP: Statistical analysis of metagenomic profiles
