# Correction to: Relationship between gut microbiota and lung function decline in patients with chronic obstructive pulmonary disease: a 1-year follow-up study

**DOI:** 10.1186/s12931-022-02093-8

**Published:** 2022-07-06

**Authors:** Yu-Chi Chiu, Shih-Wei Lee, Chi-Wei Liu, Tzuo-Yun Lan, Lawrence Shih-Hsin Wu

**Affiliations:** 1 Department of Internal Medicine, Taoyuan General Hospital, Ministry of Health and Welfare, Taoyuan, Taiwan; 2 Institute of Public Health, National Yang Ming Chiao Tung University, Taipei, Taiwan; 3 Department of Nursing, Yuanpei University of Medical Technology, Hsinchu, Taiwan; 4 Institute of Hospital and Health Care Administration, National Yang Ming Chiao Tung University, Taipei, Taiwan; 5 Graduate Institute of Biomedical Sciences, China Medical University, No. 91 Hsueh-Shih Road, Taichung, 404 Taiwan; 6 Center of Allergy, Immunology, and Microbiome (A.I.M.), China Medical University Children’s Hospital, Taichung, Taiwan

## Correction to: Respiratory Research (2022) 23:10 10.1186/s12931-022-01928-8

Following publication of the original article [1], the authors would like to correct the second affiliation information.

The second affiliation curretnly reads:

- Institute of Hospital and Health Care Administration, National Yang Ming Chiao Tung University, Taipei, Taiwan.

The correct affiliation should read as:

- Institute of Public Health, National Yang Ming Chiao Tung University, Taipei, Taiwan.

The original article [1] has been corrected.
