# Supplementary material for: Relationship between gut microbiota and lung function decline in patients with chronic obstructive pulmonary disease: a 1-year follow-up study
Source: Respir Res. 2022 Jan 15;23:10. doi: 10.1186/s12931-022-01928-8 (PMC8760664; doi:10.1186/s12931-022-01928-8)
Supplement: Supplementary file 1 — Additional file 1. Supplemental Table 1. Comparison of lung function parameters for control and decline groups. Supplemental Fig. 1. Plot of principal component analysis (PCA) for different COPD groups. S1: stage 1; S2: stage 2. Supplemental Fig. 2. The phylogenic tree of the 30 most abundant OTUs across the samples at the genus level. Supplemental Fig. 3. The heatmap analysis of the top 30 OTUs. A: At genus level. B: At species level. [file 12931_2022_1928_MOESM1_ESM.docx]

**Additional material**

Chiu *et al.* Relationship between gut microbiota and lung function decline in patients with chronic obstructive pulmonary disease: A 1-year follow-up study

**Additional Table S1.** Comparison of lung function parameters for control and decline groups

|  | pre-bronchodilator | |  | post-bronchodilator | |  |
| --- | --- | --- | --- | --- | --- | --- |
|  | Control group | Decline group | *p* value^a^ | Control group | Decline group | *p* value^a^ |
| FVC (L) |  |  |  |  |  |  |
| FVC-S1 | 2.91 ± 0.61 | 2.99 ± 0.69 | 0.641 | 3.09 ± 0.61 | 3.10 ± 0.67 | 0.966 |
| FVC-S2 | 3.09 ± 0.67 | 2.80 ± 0.70 | 0.122 | 3.17 ± 0.64 | 2.90 ± 0.69 | 0.142 |
| *p* value^b^ | 0.021 | <0.001 |  | 0.173 | <0.001 |  |
| FVC % |  |  |  |  |  |  |
| FVC %-S1 | 96.48 ± 21.05 | 107.68 ± 26.62 | 0.090 | 102.44 ± 20.04 | 111.39 ± 24.43 | 0.144 |
| FVC %-S2 | 103.04 ± 21.18 | 101.39 ± 25.56 | 0.796 | 105.52 ± 20.55 | 104.96 ± 24.08 | 0.927 |
| *p* value^b^ | 0.010 | 0.018 |  | 0.081 | 0.006 |  |
| FEV_1_ (L) |  |  |  |  |  |  |
| FEV_1_-S1 | 1.57 ± 0.61 | 1.66 ± 0.55 | 0.596 | 1.66 ± 0.59 | 1.71 ± 0.53 | 0.738 |
| FEV_1_-S2 | 1.72 ± 0.68 | 1.46 ± 0.55 | 0.124 | 1.76 ± 0.67 | 1.56 ± 0.56 | 0.235 |
| *p* value^b^ | <0.001 | <0.001 |  | 0.007 | <0.001 |  |
| FEV_1_ % |  |  |  |  |  |  |
| FEV_1_ %-S1 | 66.33 ± 24.49 | 78.89 ± 28.21 | 0.084 | 69.85 ± 23.67 | 81.68 ± 27.73 | 0.095 |
| FEV_1_ %-S2 | 73.07 ± 27.02 | 70.21 ± 26.22 | 0.692 | 74.74 ± 26.58 | 75.32 ± 27.78 | 0.937 |
| *p* value^b^ | <0.001 | <0.001 |  | 0.002 | 0.005 |  |
| FEV_1_/FVC % |  |  |  |  |  |  |
| FEV_1_/FVC %-S1 | 53.93 ± 16.60 | 55.57 ± 13.80 | 0.691 | 53.33 ± 14.20 | 55.57 ± 13.43 | 0.551 |
| FEV_1_/FVC %-S2 | 54.96 ± 15.59 | 51.93 ± 12.86 | 0.434 | 55.63 ± 15.84 | 53.89 ± 13.64 | 0.664 |
| *p* value^b^ | 0.329 | <0.001 |  | 0.039 | 0.029 |  |

^a^The statistical analysis was tested by *t* test, control group vs decline group; ^b^ The statistical analysis was tested by paired

*t* test, stage 1 vs stage 2; S1 (stage 1): lung function measurement, first time; S2 (stage 2): lung function measurement, 1-year

after stage 1.


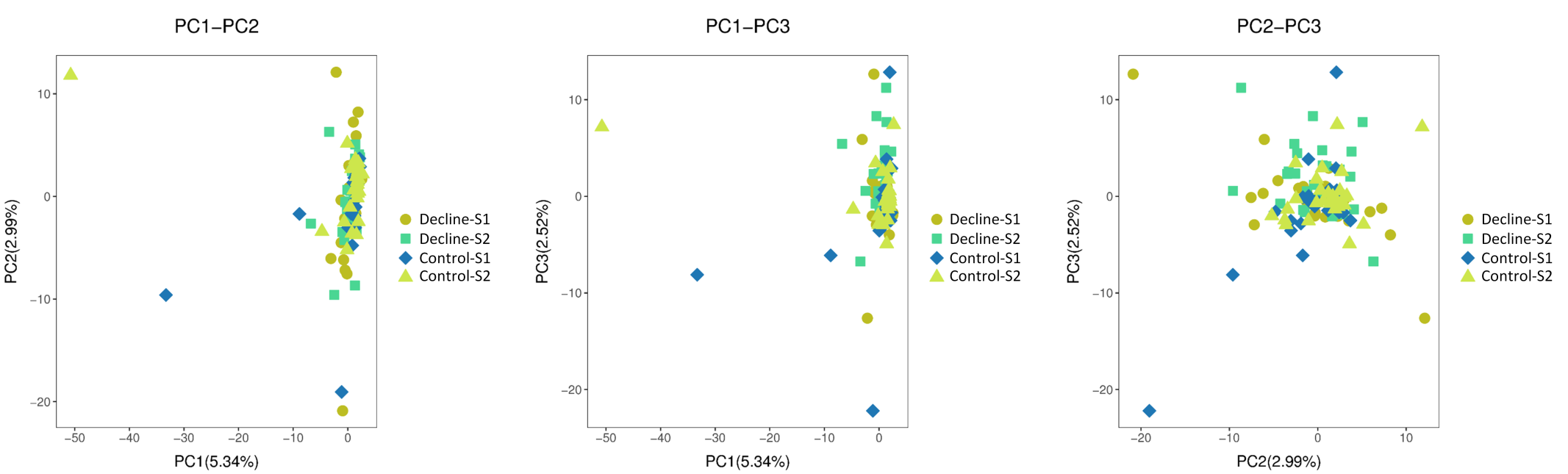


**Additional Fig. S1** Plot of principal component analysis (PCA) for different COPD groups. S1: stage 1; S2: stage 2.


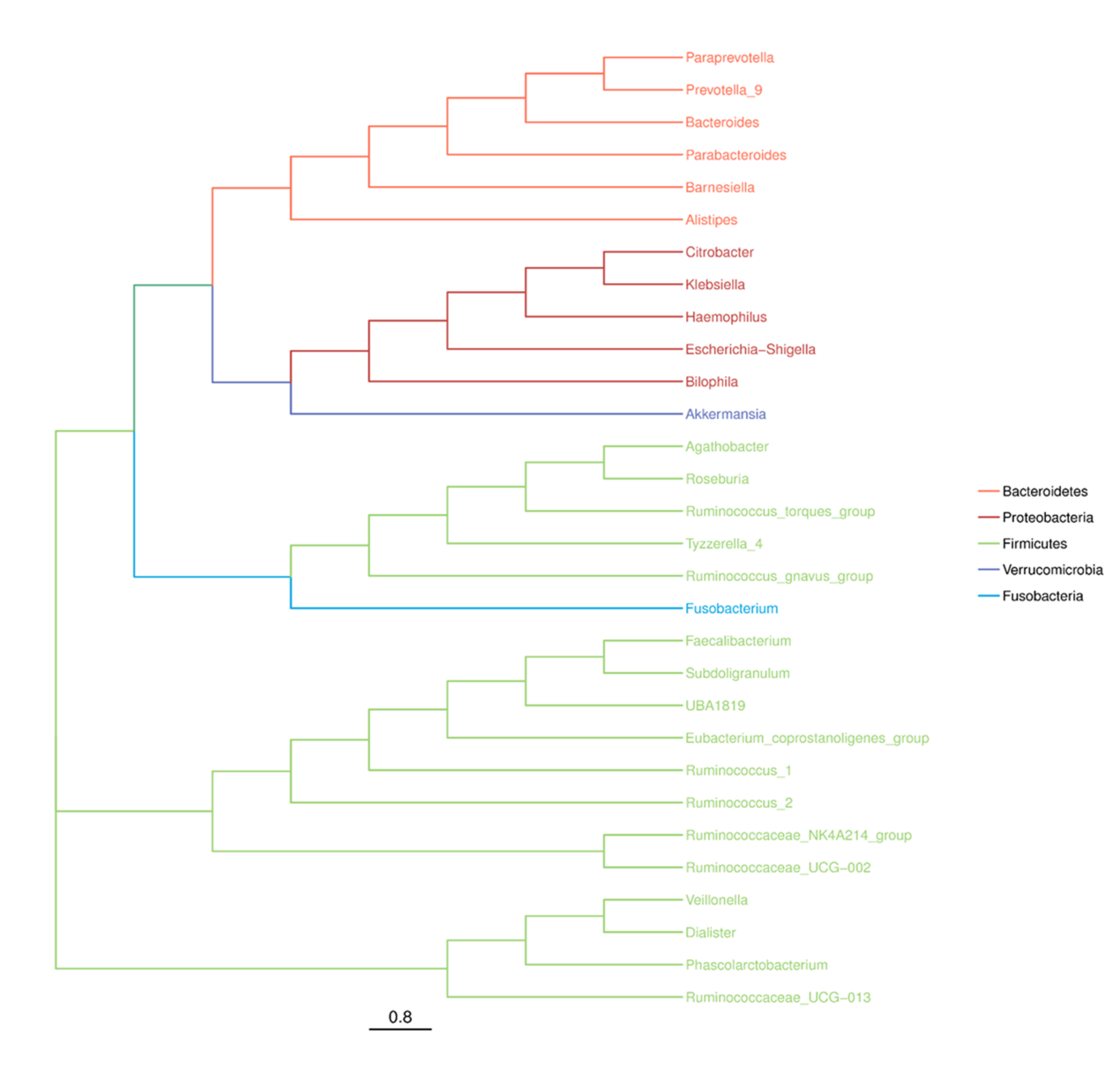
**Additional Fig. S2** The phylogenic tree of the 30 most abundant OTUs across the samples at the genus level.


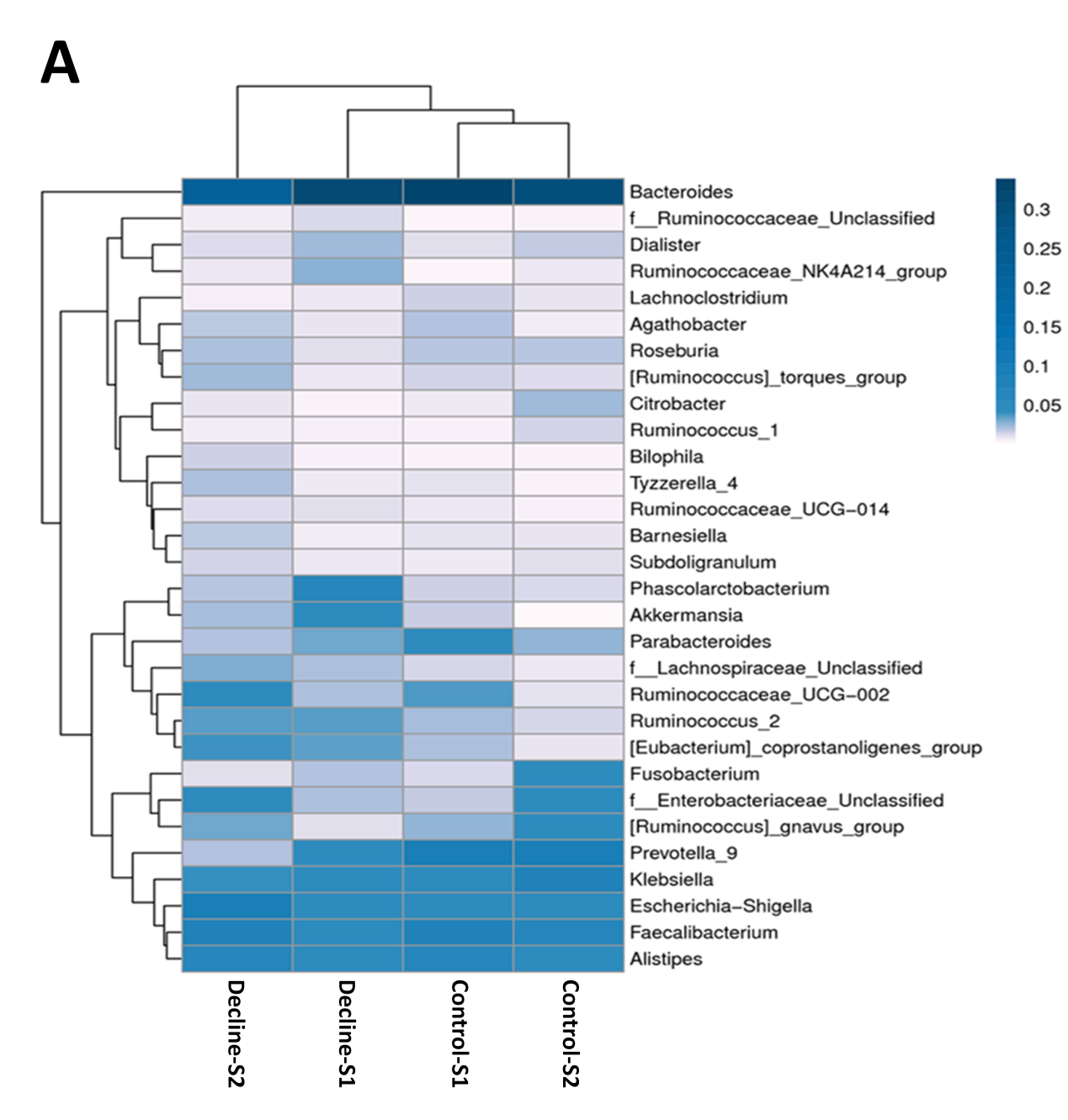


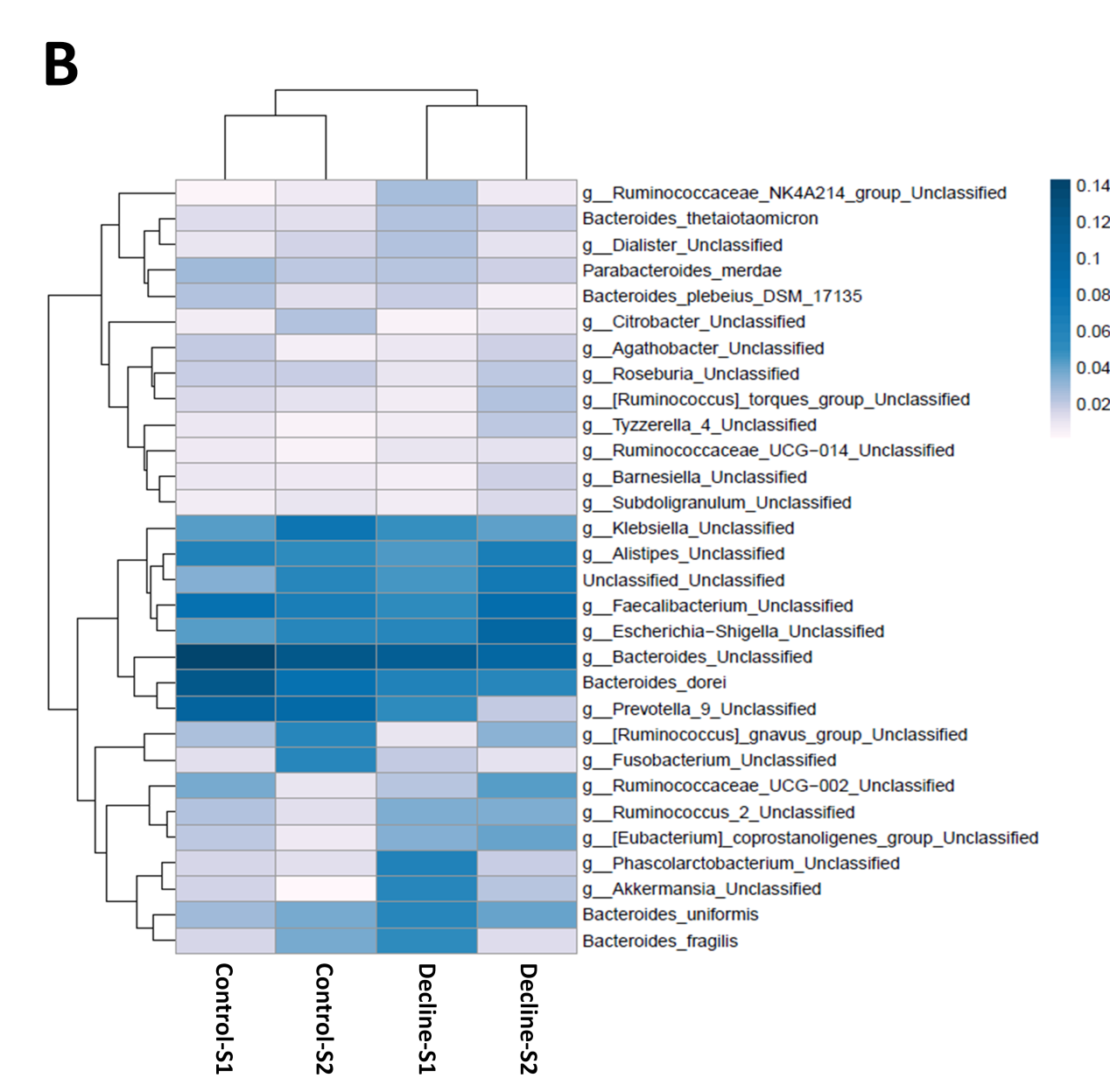


**Additional Fig. S3** The heatmap analysis of the top 30 OTUs. A: At genus level. B: At species level. The columns represent groups and the rows represent genus/species. The dendrogram above the heatmap is the cluster result of the groups and the dendrogram to the left is the genus/species cluster. The colours in the heat map represent the relative abundance of the corresponding genus in the corresponding group.

**
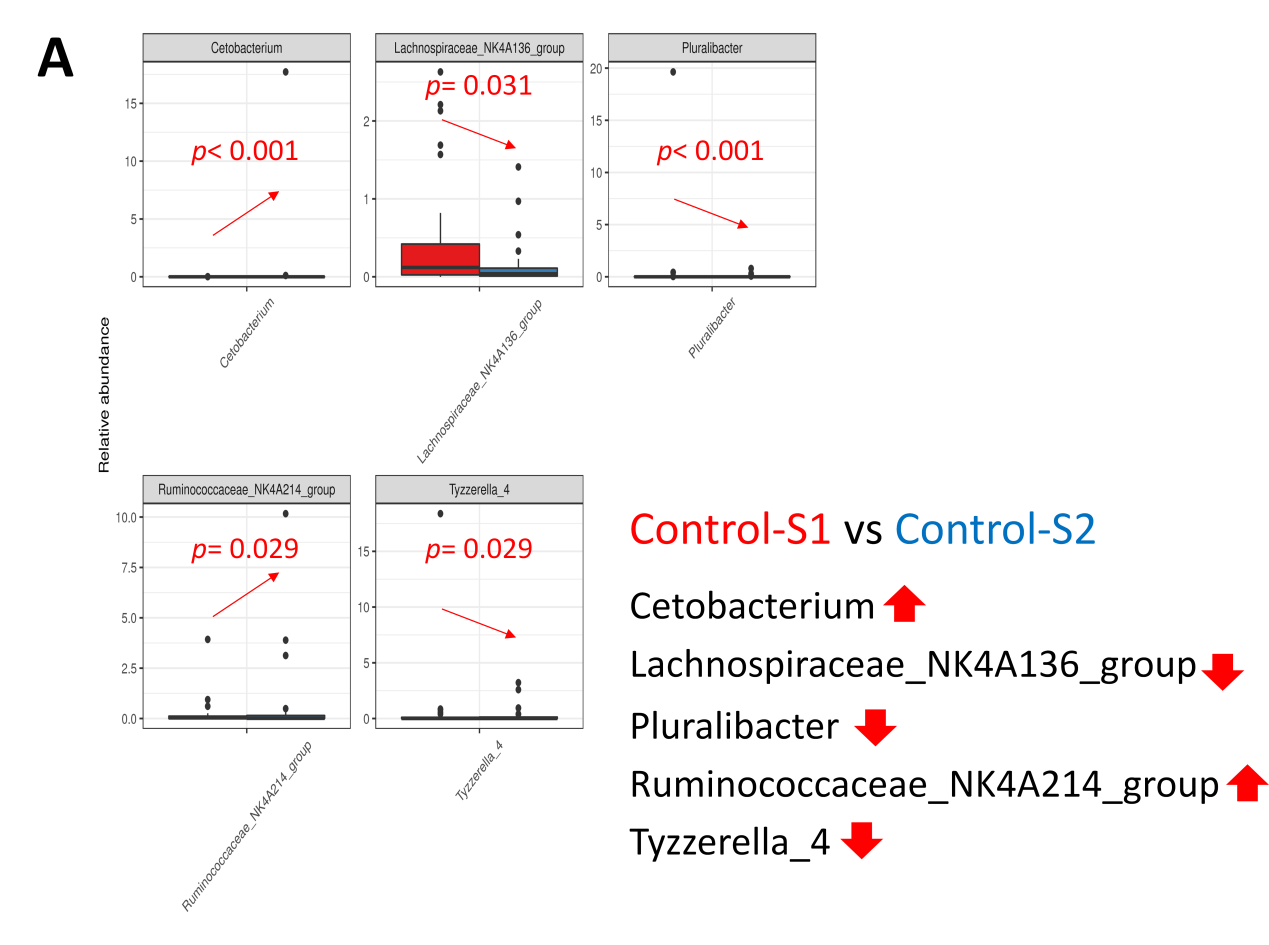
**

**
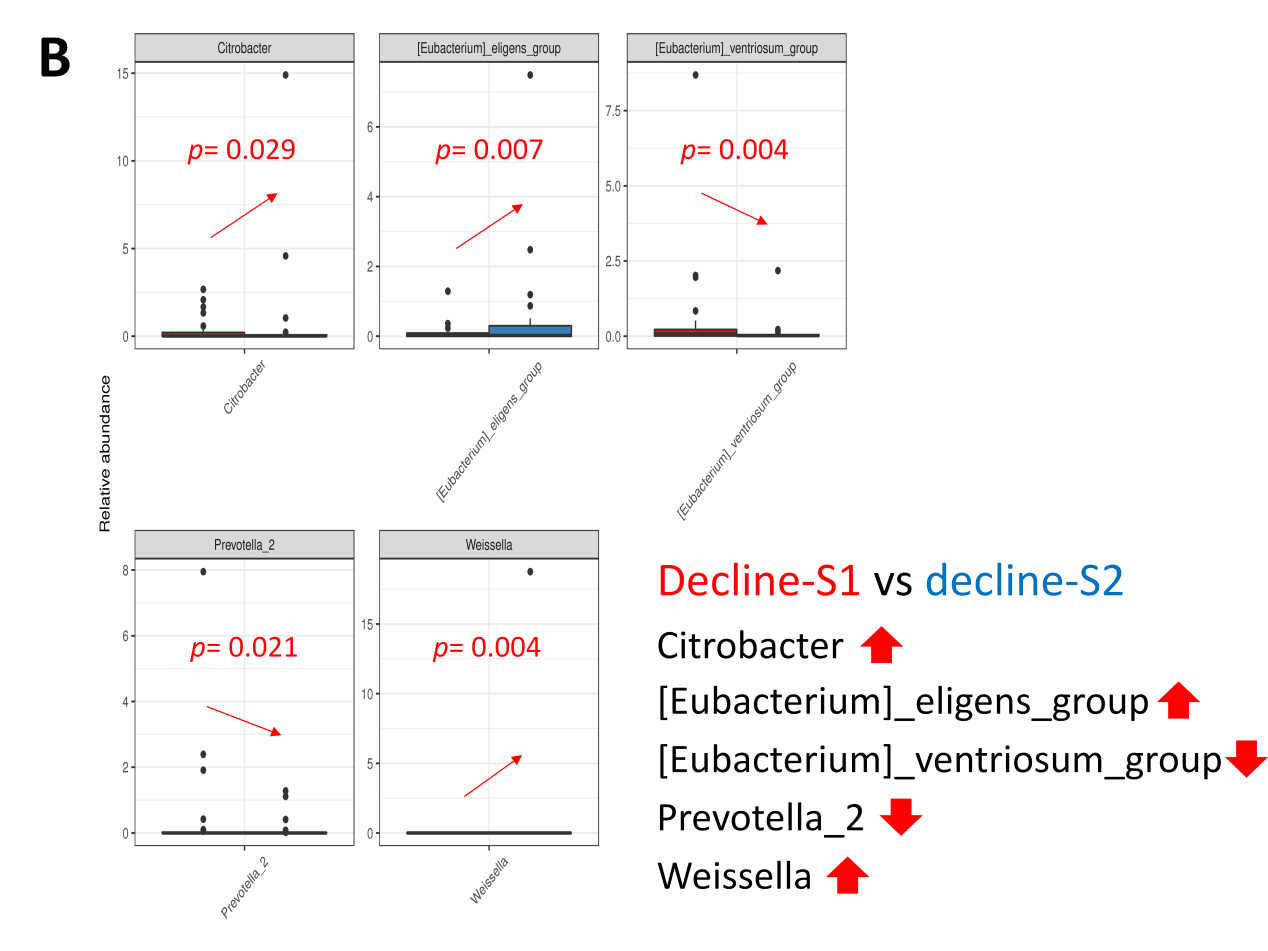
**

**
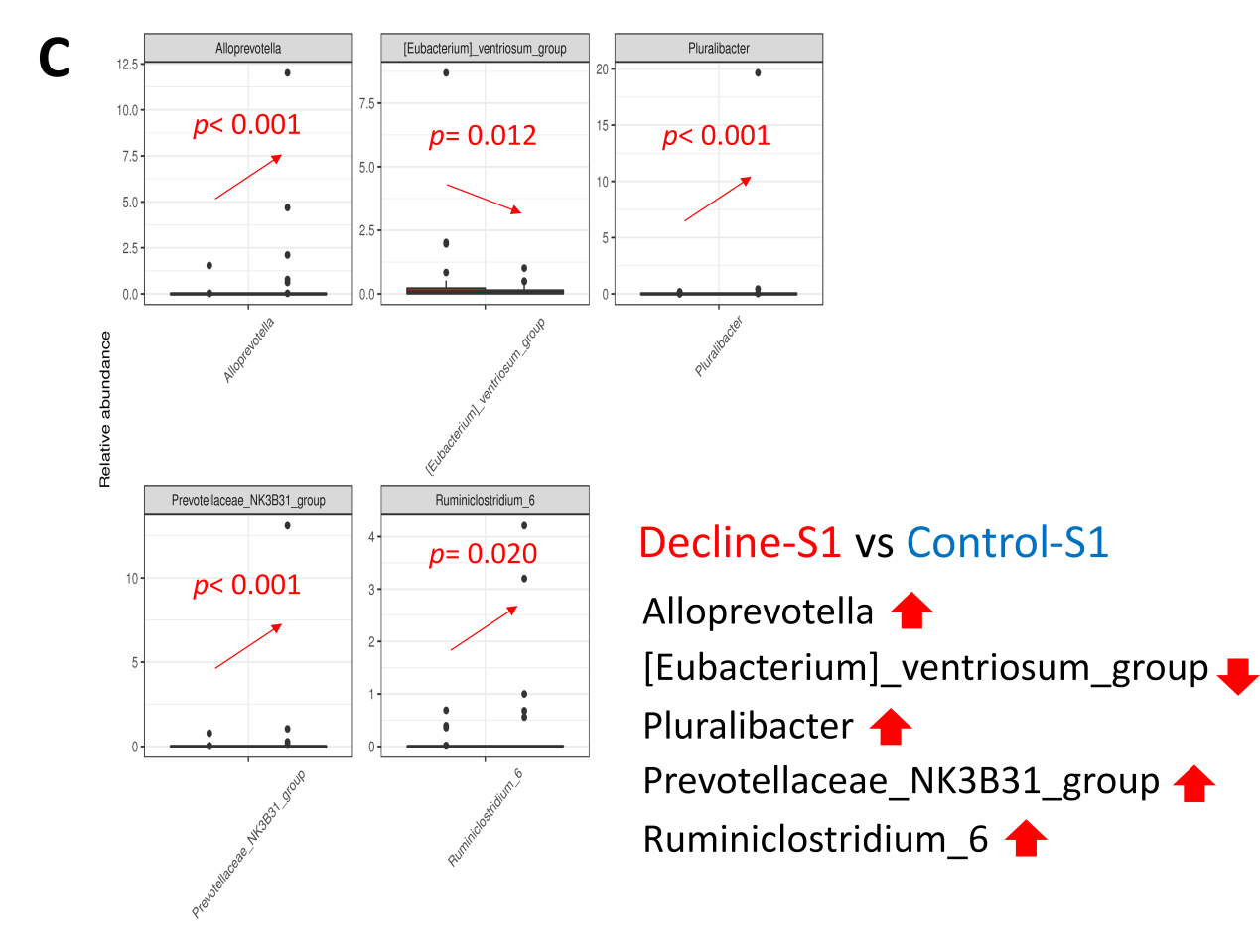
**

**
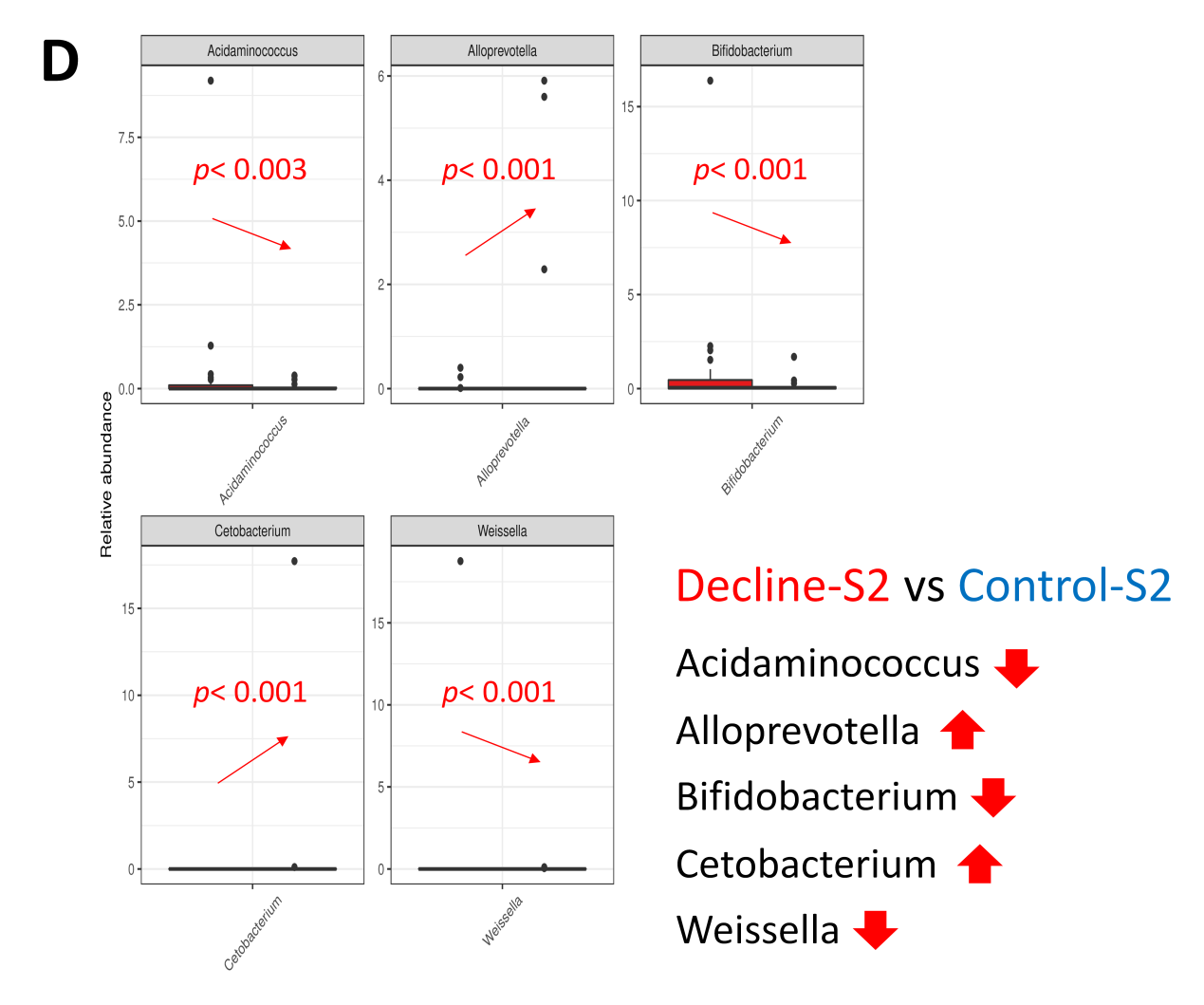
**

**Additional Fig. S4** The differences in abundance distributions of the five genera with the largest between-group differences. The X-axis indicates the names of the five genera and the Y-axis the relative abundance of each. A: Control group, stage 1 vs. stage 2; B: Decline group, stage 1 vs. stage 2; C: Stage 1, control group vs. decline group; D: Stage 2, control group vs. decline group
